# Integrative role of vitamin D related and Interleukin-28B genes polymorphism in predicting treatment outcomes of Chronic Hepatitis C

**DOI:** 10.1186/s12876-016-0440-5

**Published:** 2016-02-24

**Authors:** M. O. El-Derany, N. M. Hamdy, N. L. Al-Ansari, H. O. El-Mesallamy

**Affiliations:** Biochemistry Department, Faculty of Pharmacy, Ain Shams University, Cairo, Egypt; Endemic Medicine Department & Hepatology Unit, Faculty of Medicine, Ain Shams University, Cairo, Egypt

**Keywords:** CHC, VDR, CYP2R1, Vitamin D, G4

## Abstract

**Background:**

Improving prediction of treatment outcomes in chronic hepatitis C (CHC) genotype 4 (G4) is necessary to increase sustained viral response (SVR) rates. Vitamin D related and interferon stimulated genes are good candidates as they are recently crosstalk altering interferon response. Thus single nucleotide polymorphisms (SNPs) within some of these genes and multiple stepwise regression analysis including other independent predictors (IL28B(rs12979860), serum 25OH-vitamin D, serum alfa-fetoprotein (AFP)) were performed on a cohort of 200 Egyptian CHC patients treated with Pegylated interferon-alpha (Peg-IFN) plus ribavirin.

**Methods:**

SNPs in cytochrome P-450 (CYP2R1)(rs10741657AG), vitamin D receptor (VDR)(rs2228570AG, rs1544410CT), oligoadenylate synthetases-like (OASL)(rs1169279CT) and adenosine deaminases acting on RNA (ADAR)(rs1127309TC) genes were analyzed by real-time PCR.

**Results:**

The carrier state of A allele in VDR rs2228570 and CYP2R1 rs10741657 genes were independently associated with SVR [OR 6.453 & 3.536, *p* < 0.01 respectively]. Combining carriers of A allele in CYP2R1 and VDR genes with IL28B C/C genotype increased the probability of SVR from 80 % to reach 87.8 %, 93 % and 100 %. No relation was found between VDR rs1544410CT, ADAR rs1127309TC, OASL rs1169279CT polymorphisms and treatment outcome. Combining VDR rs2228570 A/A genotype with IL28B C/C genotype increased the probability of SVR from 82 % to reach 100 % and from 29 % to reach 80 % in C/T+ T/T IL28B genotype in none F4 liver disease patients.

**Conclusion:**

Vitamin D related (VDR rs2228570 and CYP2R1 rs10741657) and IL28B rs12979860 genes polymorphisms accurately assure SVR in naïve CHC G4 patients treated with low cost standard therapy.

## Background

Hepatitis C virus (HCV) is a hepatotropic non-cytopathic positive strand RNA virus, which represents a major cause of chronic hepatitis, cirrhosis, and hepatocellular carcinoma, affecting over 150 million people worldwide. In Egypt the condition is even worse just about every family is touched by hepatitis C it kills an estimated 40,000 Egyptian per year where at least 1 in every 10 of the population aged 15 to 59 is infected [1].

Pegylated interferon-alpha (Peg-IFN) plus ribavirin (RBV) constitutes the standard cornerstone therapy for chronic hepatitis C (CHC). Even though less than half of the patients are able to achieve sustained viral response (SVR) while the rest don’t show response or experience sever complications [2]. By the introduction of the novel direct acting antivirals (DAAs), CHC treatment modalities dramatically evolve increasing rates of SVR, treatment efficacy and tolerability especially in patients with advanced liver disease (fibrosis METAVIR score F3 or F4) [3, 4]. Yet, they are much more expensive if compared to the standard dual therapy [5] and a more questionable issue emerged about its availability in treating all CHC patients especially in low income developing countries [6]. Recently, according to the latest guidelines of the European Association for the Study of the Liver (EASL) six treatment options are available for patients infected with HCV genotype 4 (G4), including two interferon-containing regimens which are sofosbuvir and simeprevir and four interferon-free regimens which are combinations of sofosbuvir and simeprevir or sofosbuvir and ledipasvir or ombitasvir, paritaprevir and ritonavir or sofosbuvir and daily daclatasvir [4]. But EASL declared that the combination of PegIFN and RBV remains acceptable in settings where none of these options are available [4].

Thus, despite progress in DAAs there is still room for optimizing the efficacy of the lower cost standard dual therapy in naïve CHC patients. This aim could be obtained by further refining the treatment factors associated with maximal outcome benefits from dual therapy [7].

Several predictors of successful treatment response of CHC are identified [8]. Some of these depend on the virus itself others are host-related, either genetic (interleukin 28B (IL28B) rs12979860 gene polymorphism, gender and race) or acquired (insulin resistance, obesity, liver steatosis and liver fibrosis stage) [9, 10]. To refine the predictive capacity of these biomarkers for the response to Peg-IFN and RBV, additional baseline predictors of clinical response would be welcomed [10].

Vitamin D appears to possess an important immune-modulator effect beside its classical action on calcium metabolism [11]. Deficiency of vitamin D is very common in CHC patients [12]. Though, conflicting results were found by relating these deficiencies with the success of Peg-IFN and RBV therapy [13, 14]. Vitamin D undergoes two activation processes before its interaction with vitamin D receptor (VDR) [15]. The first activation is performed in the liver by cytochrome P-450 family 2, subfamily R, polypeptide 1 (CYP2R1) and produces 25-hydroxylated form of vitamin D [13]. This step produces the main circulating vitamin D form in serum. By means of CYP27B1, vitamin D is subjected to its second hydroxylation which leads to the production of 1,25(OH) active vitamin D form which interacts with its specific transmembrane receptor VDR to exert its physiological functions [16]. CYP2R1 and VDR genes have different polymorphic forms which are recently suggested to influence the efficacy of antiviral therapy. On the other hand single functional nucleotide polymorphism (SNP) of CYP27B1 gene fails to predict antiviral response in CHC patients mainly genotype 1 [7, 10].

A more recent study found that VDR crosstalk the Jak–STAT pathway through altered expression of interferon stimulated genes (ISGs) which results in calcitriol-mediated increase of hepatocellular response to interferon-alpha (IFN-α) [17]. Where, signaling the expression of ISGs by the administration of exogenous interferon provides antiviral action against HCV through interferon receptors and through Jak–STAT pathway [18]. Of these ISGs functional SNPs at oligoadenylate synthetases-like (OASL) gene and adenosine deaminases acting on RNA (ADAR) gene were found to affect the response to antiviral therapy of HCV genotype 1 [19, 20].

Knowing that different HCV genotypes respond differently to interferon antiviral therapy [21] and based on the aformetioned information we aimed to investigate for the first time the influence of SNPs in CYP2R1 gene, VDR gene, OASL gene and ADAR gene as well as IL28B gene, serum 25-OH vitamin D levels, serum levels of AFP and insulin resistance in treatment outcomes of Egyptian CHC G4 patients treated with Peg-IFN and RBV.

## Methods

### Study population

Two hundred Egyptian patient infected with HCV G4 were enrolled in this study. Baseline and clinical characteristics are summarized in Table 1. The population study included all patients with CHC G4 attended to Dr. Yassin Abdel Ghaffar center for liver diseases and researches, Cairo, Egypt who were scheduled to receive Peg-IFN and RBV therapy in accordance with the current guidelines [22]. They received Peg-IFN (Pegasys, Roche Corporation, Kenilworth, NJ or Pegintron, Roche, Basel, Switzerland) at a dosage of 180 μg per week and RBV (Copegus, Roche or Rebetol, Schering-Plough, Puerto Rico, U.S.A.) which was administered according to body weight (1000 mg/d for patients weighing <75 kg, 1200 mg/d for those weighting ≥75 kg). Patients were selected according to the following criteria: (1) older than 18 years, (2) lack of co-infection with HBV, HIV, EBV, and CMV, (3) no history of alcohol consumption, (4) no bilharzias or diabetes mellitus, (5) not suffering from other autoimmune or hematological diseases and (6) adherence to treatment. Patients were asked to stick to the follow-up visits scheduled at week 4, 12, 24, 36, 48, 72 and 6 months after end of the treatment date. Patients were defined as having a SVR if they had undetectable serum HCV-RNA (Roche Amplicor™ Assay) levels 6 months after the discontinuation of treatment. All other patients were defined as non-responders (NRs). The study was approved by the Committee on Medical Ethics of ASUH, and informed consent was obtained from each patient for participation and publishing results obtained. The study was carried out in accordance with the regulations and recommendations of the Declaration of Helsinki. Blood samples for biochemical, virologic and genetic studies were obtained during the month previous to the onset of therapy. Histopathological data were collected when a liver biopsy performed within the previous six months was available. Liver biopsy specimens were scored using the METAVIR system [23]. All biopsies were evaluated and scored by the same pathologist.

**Table 1:** Baseline clinical characteristics of HCV-infected patients included in the present study

Characteristics	SVR	NR	*P* value	Statistics (univariate)
	(*n* = 91	(*n* = 109)		OR(95%CI)
Age mean ± SD (years)	49.7 ± 7.5	51.5 ± 8.5	-	
Gender distribution[n(%)]			-	
Male	61(67)	68(62.4)		
Female	30(33)	41(37.6)		
BMI, median (range), kg/m^2^	23 ± 12	24 ± 13.5	-	
No of injections, median (range)	48(24)	48(67)	0.001	1.035(1.013-1.058)
Tolerability, [n(%)]	91(100)	88(80.7)	-	
*Adjusted No of injections ,mean ± SEM	39.23 ± 1.179	38.218 ± 1.072	-	
Viral load median, IU/mL	460,000	760,580	0.025	0.637(0.429-0.945)
400,000 IU/mL[n(%)] > Viral load	41(45.1)	38(34.9)	-	
Viral load ≥400,000 IU/mL[n(%)]	50(54.9)	71(65.1)	-	
^a^AST median (range), μkat/L	1.66(4.65)	1.44(4.22)	-	
^b^ALT median (range), μkat/L	2.04(6.22)	1.7(5.63)	-	
Albumin median (range), g/L	39 ± 20	39 ± 22	-	
Bilirubin median (range), μmol/L	13.68 ± 50.45	15.39 ± 44.46	-	
Hemoglobin median (range), g/dL	13 ± 6.1	13.5 ± 7	-	
Platelets median (range), 10^9^/L	190(241)	160(320)	0.012	3.299(1.306-8.334)
^c^TLC median (range), 10^9^/μL	6(10)	6.4(6)	-	
Cholesterol mean ± SD, mmol/L	4.41 ± 0.78	4.69 ± 0.66	-	
Glucose median (range), mmol/L	5.27 ± 3.38	5.5 ± 3.1	-	
Insulin median (range), pmol/L	79.8 ± 127	62.5 ± 147.2	-	
^d^HOMA-IR median (range)	2.4 ± 5.71	2.2 ± 6.8	-	
^e^AFP median (range), ng/mL	3.3(29.5)	7(29)	<0.001	0.165(0.071-0.383)
25-OH Vitamin D median (range),ng/mL	28 ± 38	19 ± 38	<0.001	4.877(2.512-9.468)
Cirrhosis, [n(%)]	5(5.5)	15(13.7)	-	
Fibrosis stage, [n(%)]				
F1	21(42)	12(18.4)	0.006	3.198(1.378-7.419)
F2	16(32)	29(44.6)	-	
F3	12(24)	16(24.6)	-	
F4	1(2)	8(12.3)	-	
Necroinflammatory grade, [n(%)]				
A1	26(52)	27(41.5)	-	
A2	22(44)	27(41.5)	-	
A3	2(4)	11(17)	0.04	0.204(0.043-0.97)
IL28B rs12979860 [n(%)]			Chi^2^ = 55.2 *p* < 0.001	
C/C	56(61.5)	14(12.8)	<0.001	10.857(5.379-21.913)
C/T	31(34.1)	67(61.5)	<0.001	0.324(0.181-0.579)
T/T	4(4.4)	28(25.7)	<0.001	0.133(0.045-0.395)
T allele carriers/ T allele non carriers	35/56	94/14	<0.001	0.092(0.046-0.186)

^a^ AST, aspartate aminotransferase; ^b^ ALT, alanine aminotransferase; ^c^ TLC, total leukocyte count; ^d^ HOMA-IR, homeostatic model of insulin resistance; ^e^ AFP, alfa feto protein; NR, non-responders; SVR, sustained virological responders; N, number of samples; OR, odds ratio; 95%CI Confidence Interval.; Chi-square test for genotype distribution; IL28B: interleukin 28 B

*Calculated by general linear model with adjustment for tolerability

### Laboratory analyses

Routine hematological and biochemical tests were performed after an overnight fast with the standard methods at the center’s laboratory. It includes complete blood count (CBC), serum transaminases (ALT, AST), total and conjugated bilirubin, serum albumin, alpha-feto protein (AFP), blood sugar and serum cholesterol.

### Virological testing

For all 200 patient, a baseline serum sample, collected before starting antiviral therapy, was separated and stored at _80 °C until used. The presence or absence of HCV antibodies was detemined by third generation ELISA (Dia Sorin). Serum HCV RNA was detected by using the real time polymerase chain reaction (RT-PCR) (TaqMan Roche Amplicor™ Assay) [24]. Viral load was classified as low (<400.000 IU/ml) or high (≥400.000 IU/ml), according to Wittho¨ft et al. [25].

### Genotyping

Genomic DNA was extracted with QIAamp DNA Mini Kit protocol (QIAGEN, Santa Clarita, CA) according to the manufacturer’s instructions. DNA samples were subjected to DNA quantitation and purity assessment using the NanoDrop® (ND)-1000 spectrophotometer (NanoDrop Technologies, Inc. Wilmington, USA).

### Analyses of interleukin 28B gene

Genotyping was carried out by the use of TaqMan® QRT-PCR Reagents constitutes a ready-to-use system for the detection of IL-28B rs12979860 polymorphism using (Assays-by-Design supplied by Applied Biosystems International ABI; Applied Biosystems, Foster City, CA) according to the manufacturer’s instructions.

### Analyses of Vitamin D-related genes

Genotyping was carried out by the use of TaqMan probes designed to detect the following SNPs: CYP2R1, rs10741657; VDR, rs2228570; VDR, rs1544410 (custom TaqMan SNP assay C___2958430_10; C__12060045_20 and C___8716062_10 respectively using Assays-by-Design supplied by Applied Biosystems International ABI; Applied Biosystems, Foster City, CA). The SNPs were selected on the basis of allele frequencies and functional clinical implications [7, 10]. The only nonsynonymous SNP confirmed for the VDR gene was included in the study. No other nonsynonymous SNPs have been described to occur in Caucasian individuals with minor allele frequencies over 0.001.

### Analysis of interferon stimulated genes

Genotyping was carried out by the use of TaqMan probes designed to detect the following SNPs: OASL rs1169279 and ADAR, rs1127309 (custom TaqMan SNP assay C___1263582_10 and C____131814_1_; using Assays-by-Design supplied by Applied Biosystems International ABI; Applied Biosystems, Foster City, CA). The SNPs were also selected on the basis of allele frequencies and functional of clinical implications [19, 20] (see the website http://www.ncbi.nlm.nih.gov/projects/SNP/).

Putative departures of Hardy-Weinberg Equilibrium was calculated by using the software Haploview 4.1. [26].

### Serum 25-OH vitamin D and serum insulin

Serum human 25-OH vitamin D levels were determined by enzyme linked immunosorbent assay (ELISA) using commercially available kits (DRG International, Inc., USA). The data are expressed as nanograms per milliliter. Serum insulin was also quantified using ELISA technique using commercial available kit (Nova Tec Immundiagnostica GmbH, Germany). The homeostasis model assessment of insulin resistance (HOMA-IR) was calculated from fasting insulin and FBG by the following equation: HOMA-IR = fasting insulin (μU/mL) × FBG (mg/dL)/405 [27]. All ELISA procedures were done by Hyprep Automated ELISA system (Hyperion Inc, Miami, FL) according to the manufacturer’s instructions.

### Statistical analysis

IBM SPSS statistics (V. 22.0, IBM Corp., USA, 2013) was used for data analysis. Shapiro-Wilk test was used to test the normal distribution of data. Continuous variables were expressed as mean ± SD for parametric data while median (range) was used for non- parametric data. Additionally, categorical variables were presented as frequencies (percentage). Comparison between two independent mean groups for parametric data were performed using Student’s *t*-test and Wilcoxon rank-sum test was carried out for comparison between two independent groups for non-parametric. The associations between categorical variables were evaluated using a Pearson chi-squared test and, when appropriate, a chi-squared test for linear trend. Hardy–Weinberg equilibrium was assessed in the study population. General linear model was used to control for potential confounders. Significant covariates at binary logistic regression analysis were included in a multivariate stepwise logistic regression model with a forward approach to identify independent predictors of SVR. In addition, any skewed data were logarithmically transformed before performing simple and multiple binary stepwise regression analyses. The AUROC indicated the prediction capacity of this analysis. The results were reported as odds ratio (OR) and their 95 % confidence intervals (CIs). A difference of *p* <0.05 was considered significant.

## Results

The clinical and demographic characteristics of the 200 patient are shown in Table 1. 54.5 % of the patients (*n* = 109) were classified as NR and 45.5 % (*n* = 91) were classified as SVR. The univariate analysis expressed in Table 1 revealed the following variables were significantly linked with SVR rates: lower viral load (OR = 0.637–95 % CI =0.429–0.945- *p* = 0.025), higher platelets count (OR = 3.299–95 % CI =1.306–8.334- *p* = 0.012), lower serum AFP levels (OR = 0.165–95 % CI =0.071–0.383- *p* < 0.001), higher serum 25-OH vitamin D levels (OR = 4.877–95 % CI =2.512–9.468- *p* < 0.001), the absence of higher fibrosis grade (OR = 3.198–95 % CI =1.378–7.419- *p* = 0.006), and the absence of A3 necroinflammatory grade (OR = 0.204–95 % CI =0.043–0.97- *p* = 0.04). Additionally, number of injections was found to be related to SVR with (OR = 1.035–95 % CI =1.013–1.058- *p* = 0.001). General linear model was performed to control confounding variables as (age, gender, BMI and interferon tolerability) and found that all adjusted values for variables were still significantly related to treatment response except for the adjusted number of injections which loses its significance to SVR due to the effect of interferon tolerability where number of injections were found to be heavily dependent on tolerability with *p* < 0.001 rather than on treatment response with *p* = 0.204.

Exploring the association of IL28B rs12979870, CYP2R1 rs10741657, VDR (rs2228570, rs1544410), OASL rs1169279 and ADAR rs1127309 genes polymorphism with response to antiviral treatment were shown in Tables 1 and 2, it was found that the absence of IL28B rs12979870T allele carrier state (chi^2^ = 51.69- *p* < 0.001), the prescence of CYP2R1 rs10741657 and VDR rs2228570 A allele carrier states (chi^2^ = 4.888- *p* = 0.027, chi^2^ = 4.138- *p* = 0.042 respectively), and OASL rs1169279T allele carrier state (chi^2^ = 6.23- *p* = 0.013), were significantly predicting SVR in CHC G4 patients.

**Table 2:** Association analysis of genes polymorphisms with the response to antiviral treatment

Genotype/ Allele	SVR	NR	*P* value	OR(95%CI)
	*N* = 91	*N* = 109		
CYP2R1 rs10741657 [n(%)]			Chi^2^ = 5.38 *p* = 0.06	
A/A	7(7.7)	4(3.7)		
A/G	44(48.4)	40(36.7)		
G/G	40(44)	59.6))65	0.028	0.531(0.302-0.933)
A allele carriers/A allele non carriers	51/40	44/65	0.027	1.884(1.072-3.310)
VDR rs2228570 [n(%)]			Chi^2^ = 8.66 *p* = 0.013	
A/A	13(14.3)	4(3.7)	0.013	4.375(1.374-13.932)
A/G	36(39.6)	39(35.8)		
G/G	42(46.2)	66(60.6)		
A allele carriers/A allele non carriers	49/42	43/66	0.042	1.791(1.019-3.146)
VDR rs1544410 [n(%)]			n.s.	
C/C	30(33)	38(34.9)		
C/T	39(42.9)	47(43.1)		
T/T	22(24.9)	24(22)		
T allele carriers/ T allele non carriers	61/30	71/38		
OASL rs1169279 [n(%)]			Chi^2^ = 5.4 *p* = 0.06	
C/C	19(20.9)	39(55.8)	0.022	0.474(0.250-0.898)
C/T	52(57.1)	49(45)		
T/T	20(22)	21(19.3)		
T allele carriers/ T allele non carriers	72/19	70/39	0.013	2.26(1.182-4.318)
ADAR rs1127309 [n(%)]			n.s.	
C/C	30(33)	38(34.9)		
C/T	39(42.9)	47(43.1)		
T/T	22(24.2)	24(22)		
T allele carriers/ T allele non carriers	61/30	71/109		

OR, odds ratio; 95%CI Confidence Interval; n.s., non-significant; SVR: sustained viral response; NR: non response; CYP2R1: cytochrome P-450 family 2, subfamily R; VDR: vitamin D receptor; OASL: oligoadenylate synthetases-like; ADAR: adenosine deaminases acting on RNA

Chi-square test for genotype distribution

These significant variables were included in the multivariate binary logistics regression analysis after controlling for the confounders. Where, IL28B gene polymorphism showed the highest significance as C/T + T/T genotypes were inversely associated with SVR (OR = 0.064–95 % CI =0.022–0.184- *p* < 0.001). Beside serum AFP levels and basic viral load were found to be inversely related to SVR (OR = 0.241–95 % CI =0.071–0.816- *p* = 0.022) and (OR = 0.523–95 % CI =0.228–0.951- *p* = 0.033), respectively. On the other hand, serum 25-OH vitamin D levels were confirmed by this study to predict SVR (OR = 7.361–95 % CI =2.828–19.162- *p* < 0.001). A negative cut-off value for serum 25-OH vitamin D levels was calculated to be up to 12 ng/ml with a negative predictive value of 87.5 % and sensitivity of 95.6 %. While a positive predictive value of 69.6 % and specificity of 93.6 % was calculated for the positive cut-off value to be up to 38 ng/ml.

Additionally, the carrier state of allele A at CYP2R1 rs10741657 was found to be inversely related to the probability of therapeutic failure (OR = 3.536–95 % CI = 1.444–8.659- *p* = 0.006). Besides, the carrier state of the minor A allele at VDR rs2228570 was significantly related to the probability of obtaining SVR (OR = 6.453–95 % CI =2.348–17.739- *p* < 0.001).

**Fig. 1:**
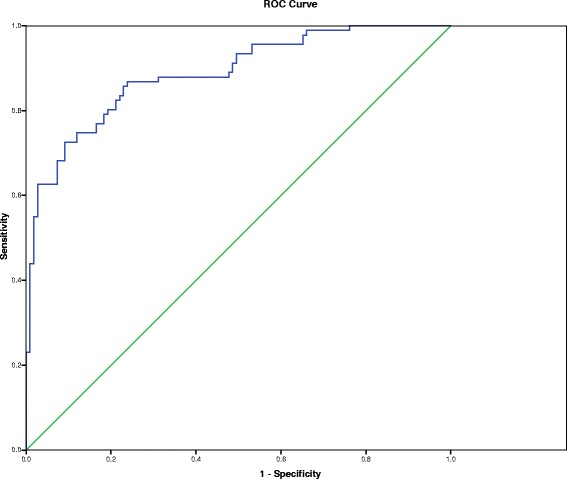
ROC curve of the multivariate analysis. Area under the curve (AUC) was 0.887 (95 % CI = 0.842–0.993)

The ROC was plotted in accordance with the same model as expressed in Fig. 1. The predictive capacity of the final model was quite good as indicated by the AUROC 0.887(95 % CI = 0.842–0.993). Multiple stepwise logistic regression analysis was made to establish the increase in the predictive value of the well-known and recognized IL28B rs12979870 C/T polymorphism. It showed that our model presented significantly increase the sensitivity of IL28B rs12979870 from 61.5 % to reach 75.8 % as well as increasing the accuracy of IL28B rs12979870 from 75.5 % to reach 81 % without significantly affecting IL28B rs12979870 specificity to be 85.3 %.

For confirming the integrative role of VDR rs2228570, CYP2R1 rs10741657 polymorphisms with IL28B rs12979870 polymorphism we further classified our SVR data into four groups depending on IL28B genotype regarding the carrier state of both CYP2R1 rs10741657 and VDR rs2228570 gene. Based on our results the rate of SVR was predicted by 80 % of IL28B C/C genotype patients. We confirmed for the first time that the combination of A allele carrier of VDR rs2228570 gene with IL28B C/C genotype increases SVR rate to reach 93 % and 87.8 % for A allele carrier of CYP2R1 rs10741657 gene with IL28B C/C (*P* < 0.001). Additionally, for each examined variable it was found that there is a highly significant linear trend in the decrease of the SVR rate as shown in Table 3. Formulating a model combining A allele carrier of VDR rs2228570 and CYP2R1 rs10741657 gene and IL28B C/C genotype increased the SVR rate reaching 100 % and 17 % in non A allele carrier of VDR rs2228570 and CYP2R1 rs10741657 gene with IL28B C/T + T/T genotype (*P* < 0.001).

**Table 3:** Rates of SVR in relationship to IL28B rs12979860 and vitamin D related genes polymorphisms

	SVR				*P* value
IL28B rs12979860	C/C	C/C	C/T + T/T	C/T + T/T	
CYP2R1 rs10741657	A allele carrier	Non A carrier	A allele carrier	Non A carrier	
N(%)	29/33 (87.8 %)	27/37(72.9 %)	22/62(35.4 %)	13/68(19.1 %)	<0.001
VDR rs2228570	A allele carrier	Non A carrier	A allele carrier	Non A carrier	
N(%)	27/29(93 %)	29/41(70.7 %)	23/66(34.8 %)	12/64(18.7)	<0.001
CYP2R1 & VDR	A allele carrier	Non A carrier	A allele carrier	Non A carrier	
N(%)	17(100 %)	15/23(65.2 %)	15/30(50 %)	6/35(17 %)	<0.001

The statistical analysis was performed using a chi-squared test for linear trend

IL28B: Interleukin 28 B; SVR: sustained viral response; CYP2R1: cytochrome P-450 family 2, subfamily R; VDR: vitamin D receptor

Interestingly we investigate the role of our predictors in different fibrosis grade models. Actually liver biopsies were available for 115 patient. So 106 patient remained after excluding advanced fibrosis F4 and cirrhotic patient at which 53.4 % (*n* = 57) were NR and 46.2 % (*n* = 49) were SVR as shown in Table 4. Univariate analysis showed that some variables significantly predict SVR as: higher serum 25-OH vitamin D levels (OR = 1.068–95 % CI =1.024–1.115- *p* = 0.002), the absence of IL28B rs12979870T allele carrier state (chi^2^ = 26.281- *p* < 0.001) and the presence of VDR rs2228570 A/A genotype state (chi^2^ = 8.511- *p* = 0.018). CYP2R1 rs10741657 A allele carrier state showed insignificant relation to SVR (chi2 = 2.834- *p* = 0.12). Number of injections was found to be related to SVR (OR = 1.042–95 % CI =1.013–1.072 - *p* = 0.004) and lower serum AFP levels (OR = 0.563–95 % CI =0.342–0.928- *p* = 0.024) yet the adjusted number of injections for interferon tolerability and the adjusted serum AFP levels also lost their significance to SVR with *p* = 0.386 and *p* = 0.205 respectively.

**Table 4:** Baseline clinical characteristics of HCV-infected F1, F2 and F3 patients

Characteristics	SVR	NR	*P* value	Statistics (univariate)
	(*n* = 49)	(*n* = 57)		OR(95%CI)
Age mean ± SD (years)	50.2 ± 8.5	49.3 ± 7.9	-	
Gender distribution[n(%)]			-	
Male	34(69.3)	34(59.6)		
Female	15(30.6)	23(40.3)		
BMI, median (range), kg/m^2^	23 ± 12	23 ± 12	-	
No of injection,s median (range)	48(24)	24(62)	0.004	1.042 (1.013- 1.072)
Tolerability, [n(%)]	49(100)	43(75.4)		
*Adjusted No of injections ,mean ± SEM	37.28 ± 1.74	35.14 ± 1.63	-	
Viral load median, IU/mL	432,210	738,902	-	
^a^AST median (range), μkat/L	1.66(4.23)	1.28(4.2)	-	
^b^ALT median (range), μkat/L	1.78(5.47)	2.091(5.62)	-	
Albumin median (range), g/L	39 ± 20	39 ± 10	-	
Bilirubin median (range), μmol/L	15.39 ± 50.44	13.6 ± 25.6	-	
Hemoglobin median (range), g/dL	13 ± 5.6	13.8 ± 4.6	-	
Platelets median (range), 10^9^/L	163(236)	170(207)	-	
^c^TLC mean ± SD, 10^9^/μL	6.5(1.7)	6.5(1.3)	-	
Cholesterol mean ± SD, mmol/L	4.1 ± 0.86	4.2 ± 0.6	-	
Glucose median (range), mmol/L	5.27 ± 3.277	5.5 ± 3.1	-	
Insulin median (range), pmol/L	79.6 ± 113.2	63.5 ± 133.3	-	
^d^HOMA-IR median (range)	2.5 ± 5.71	2.16 ± 6.04	-	
^e^AFP median (range), ng/mL	3.5(29.5)	8(26.3)	0.024	0.563 (0.342- 0.928)
*Adjusted AFP, mean ± SE	3.5 ± 0.049	3.448 ± 0.045		
25-OH Vitamin D mean ± SD, ng/mL	27.3 ± 9.2	21.07 ± 10.1	0.002	1.068(1.024-1.115)
IL28B rs12979860 [n(%)]			Chi^2^ = 31.35 *p* < 0.001	
C/C	28(57.1)	6(10.5)	<0.001	11.33(4.09-39.35)
C/T	20(40.8)	35(62.4)		
T/T	1(2)	16(28.07)		
T allele carriers/ T allele non carriers	21/28	51/6	<0.001	0.088(0.032-0.244)
VDR rs2228570 [n(%)]			Chi^2^ = 8.554 *p* = 0.014	
A/A	9(18.3)	1(1.7)	0.018	12.6(1.53-103.4)
A/G	18(36.7)	24(42.1)		
G/G	22(44.8)	32(56.1)		

^a^ AST, aspartate aminotransferase; ^b^ ALT, alanine aminotransferase; ^c^ TLC, total leukocyte count; ^d^ HOMA-IR, homeostatic model of insulin resistance; ^e^ AFP, alfa feto protein; NR, non-responders; SVR, sustained virological responders; N, number of samples; OR, odds ratio; 95%CI Confidence Interval.; Chi-square test for genotype distribution; IL28B: interleukin 28 B

*Calculated by general linear model with adjustment for tolerability

Multivariate binary logistic regression analysis were made after controlling for the confounders and found that IL28B gene polymorphism showed highest significance as C/T + T/T genotypes were inversely associated with SVR (OR = 0.138–95 % CI =0.045–0.423- *p* = 0.001). While, serum 25-OH vitamin D levels (OR = 1.076–95 % CI =1.013–1.144- *p* = 0.018) and also the presence of A/A genotype state of VDR rs2228570 (OR = 10.3–95 % CI =1.118–94.887- *p* = 0.04) were directly associated with SVR. The ROC was plotted in accordance with the same model showed that the AUROC was 0.837 (95 % CI = 0.76–0.951). Multiple stepwise logistic regression analysis showed that our model significantly increase the sensitivity of IL28B rs12979870 from 57.1 % to reach 67.3 % as well as increasing the accuracy of IL28B rs12979870 from 74.5 % to reach 77.4 % without significantly affecting IL28B rs12979870 specificity to be 89.5 %.

Classifying our SVR data into four groups depending on IL28B genotype found that combining IL28B C/C genotype with VDR rs2228570 A/A genotype increased the predictive capacity of IL28B C/C genotype from 82.2 % to reach 100 %. More importantly combining VDR rs2228570 A/A genotype with IL28B C/T + T/T increased the SVR from 29 % to reach 80 %.

By formulating a second model excluding F3, F4 and cirrhotic patients 78 patient remained at which 52.5 % (*n* = 41) were NR and 47.4 % (*n* = 37) were SVR. It was found that IL28B C/C genotype is the only predictor of SVR (OR = 20.81–95 % CI =5.394–80.286 - *p* < 0.001). The AUROC equals 0.774 (95 % CI = 0.665–0.883) the calculated specificity, sensitivity and accuracy were 92.7 %, 62.2 % and 78.2 % respectively.

## Discussion

The need for optimizing the standard dual anti-HCV therapy is mandatory owing to the high socioeconomic burden associated with recently introduced DAAs [6, 28]. Although DAAs showed strong anti-HCV effects, DAA monotherapy induced drug-resistant and virus mutations. Concluding that the main role of DAAs has been to increase the treatment efficacy of Peg-IFN-α and RBV [29]. This paves the way for elucidating new markers that would improve the prediction of Peg-IFN and RBV treatment outcomes. Although several studies have identified specific viral and host genetic factors that predict treatment outcomes, studies regarding G4 are few and the pursuit of further surrogate factors able to optimize therapeutic regimes remains challenging [30]. Due to these facts, we have a great research interest in examining for the first time the relationship between previously described polymorphisms of vitamin D related genes and ISGs with the treatment of HCV G4 patients.

In this study we found a predictive potential of baseline 25-OH vitamin D levels on treatment outcomes. Recent findings from various studies investigating the predictive role of vitamin D levels offered conflicting evidences. On one hand low pretreatment vitamin D concentrations correlated with poor responsiveness to antiviral therapy [31] and on the other hand Kitson et al., showed that vitamin D levels seemed unable to predict treatment response in patients infected with HCV genotype 1 [14]. Yet all these hitherto existing analyses were made on genotype 1 with incomplete consideration of genetic factors affecting vitamin D levels. In this present study only G4 was included and the effect of SNP’s within major regulatory enzyme and in vitamin D receptor, which were previously shown to affect vitamin D concentrations, activity and were associated with outcomes of antiviral therapy, were considered [7, 10, 32].

Our results revealed that high serum 25-OH vitamin D levels in the multivariate analysis that included the most potent predictors of viral response at baseline increase the SVR of Peg-IFN and RBV treatment. Also our study concluded that the negative predictive value was 12 ng/ml for 87.5 % of CHC G4 patients who fails to reach SVR and the positive predictive value of 69.6 % was found to be 38 ng/ml which weren’t calculated for the Egyptian G4 patients before yet found in agreement to other studies [33]. Interestingly high serum 25-OH vitamin D levels predicted SVR in noneF4 fibrosis grade patients which was recently emphasized by previous studies showing that vitamin D deficiency is associated with advanced liver disease and is an indicator for a poor outcome [34, 35]. This highlights the importance of vitamin D in disease worsening via fibrosis progression and thus it reflects indirectly the importance of fibrosis resolution in CHC treatment [36].

Additionally, the analysis of an already identified SNP (rs10741657) within the CYP2R1 gene on chromosome 11p15, which is one of the main polymorphisms found to be associated with 25(OH) vitamin D circulating levels [37], showed that the carrier state of allele A at CYP2R1 gene predicts SVR. This comes in accordance with a recent study that focused on the association of the absence of A allele with vitamin D insufficiency [38]. Where, our study confirmed that the presence of A allele is associated with higher vitamin D serum levels and is independently predicting SVR in multivariate logistic regression analysis yet it failed to predict response in noneF4 liver disease patients this might be attributed to small sample size.

Moreover, studying the common nonsynonymous SNP in the VDR gene (rs2228570) which cause a threonine-metionine change in the VDR, found that those patients carrying the minor A allele in homo- or heterozygosis obtained SVR at a higher rate than patients with the rs2228570 G/G genotype. This agrees with a recent study made on genotype 1 [10]. Our study additionally showed for the first time that VDR rs2228570 A/A genotype is an independent predictor of SVR in noneF4 fibrosis grade CHC G4 patients which adds to the importance of this rs2228570 in predicting treatment success. In contrast we found no association between SNP in VDR gene (rs1544410) and treatment outcome in our population. This can be explained as our study include only single rs1544410 alone unlike others [10, 39] who categorize the patients as non-carriers/carriers of the rs1544410C- rs7975232C - rs731236A three allelic combination. Thereby, we concluded that we can’t account on VDR gene rs1544410 SNP alone in predicting therapeutic failure.

Moreover, a recent study highlighting the possible interaction between VDR and ISGs suggested a role for vitamin D in the response to IFN-α–based therapy [17]. Importantly, and in line with these observations, our study assayed two functional SNPs at two important ISGs which were proven by previous studies to predict antiviral response of CHC genotype 1 [19, 20]. First one was OASL being recognized as a significant molecule in antiviral response with the 2′-5′ oligoadenylate catalytic activity in innate viral clearance. The SNP (rs1169279) is located in the 5′ upstream region of the gene and found to be a promoter polymorphism which may regulate gene expression. Our findings identified that OASL gene polymorphism is not the main player in HCV G4 treatment response as T allele carriers lost their significance in multivariate regression analysis. This can be attributed to the difference in viral genotypes as the previous study was made on genotype 1 [19]. Additionally, it also showed no relation in none-advanced liver disease patients though, larger sample size is recommended to assure our findings.

Regarding the second ISG examined SNP (rs1127309) is a synonymous variant in ADAR gene, our study on the whole populatin and after excluding advanced fibrosis grades patients found no association between ADAR gene polymorphism and Peg-IFN and RBV treatment outcomes. This disagrees with the previous study made by Welzel, et al. [20]. And could be explained by the difference in the percent of genetic variation (presence of T allele) accounted by the selected ADAR SNP. Where in European American the presence of T allele is about (50 %) and our Caucasian population is (66 %). Additionally, the previous study was a Hepatitis C Antiviral Long-term Treatment against Cirrhosis (HALT-C) trial unlike our study which includes insignificant number of cirrhotic patients.

In agreement with other studies suggested adding AFP to the list of predictive factors for treatment response in CHC G4 [40, 41]. Our results showed that increased AFP serum levels are an independent factor of therapeutic failure. This may be explained by intense hepatic progenitor cells expression in NR compared to SVR. Hepatic progenitor cells arise in the periportal region of the liver and may be responsible for liver regeneration and they express high levels of AFP. Quite interestingly, hepatic progenitor cells expression is associated with response to treatment, being higher in NR compared with responders. This may postulate the factor explaining the association of AFP with therapeutic failure [42, 43]. Our results showed that serum AFP levels is not a predictor of SVR in none-advanced liver disease and this also agreed with a study made on G4 [41].

In alignment with other published studies [30, 44] our research didn’t find association between cholesterol levels, insulin levels and HOMA-IR with antiviral response this may be probably explained by the absence of diabetes among our patients. On the other hand it was found that baseline lower viral load is a direct predictor to SVR rates as proved previously [45].

Finally we confirmed for the first time that vitamin D related genes (VDR rs2228570 and CYP2R1 rs10741657) polymorphism are completely independent of each other, may be usefully combined to enhance the predictive ability for treatment response. Where combing these two genes with IL28B rs12979860 C/C genotype increase the probability of SVR from 80 % to reach 100 %. Compared with patients carrying the IL28B rs12979860 C/C genotype and who carry at least one A allele of VDR rs2228570 and CYP2R1 rs10741657, the none A allele carriers identified patients showed a lower probability of SVR attainment. Furthermore, carrying at least one T allele of IL28B along with none A allele carrier of VDR rs2228570 and CYP2R1 rs10741657 gene showed the lowest probability of attaining the same viral endpoint. Yet increased the probability of SVR in IL28B C/T + T/T from 27 % to reach 50 %. Advanced fibrosis was proven previously to be a main factor for Peg-IFN and RBV treatment failure [45]. Thus more practically our study interestingly found that only serum levels of 25-OH vitamin D, VDR rs2228570 A/A genotype together with IL28B rs12979860 C/C genotype were the only predictors of SVR in none-F4 CHC patients. Moreover, we proved that the presence of VDR rs2228570 A/A genotype and IL28B rs12979860 C/C genotype increased SVR rates from 82 % reaching 100 % and more importantly combining VDR rs2228570 A/A genotype and IL28B C/T + T/T genotypes dramatically increase SVR from 29 % to reach 80 % which impressively increase treatment success prediction. Although promising yet larger sample size is required to assure our findings.

However in accordance with these data presented vitamin D related genes play an integrative role with IL2B gene in achieving SVR rates in CHC G4 patients.

## Conclusions

In conclusion two polymorphic sites in VDR gene and CYP2R1 gene were found to increase the sensitivity and accuracy of IL28B gene in predicting SVR rates for Peg-IFN and RBV therapy in CHC patients G4. The association of these polymorphisms with the response rate is independent from other well-known predictors of response at baseline that were included in the multivariate analysis as potentially confounding factors. The use of these new genes could facilitate the treatment decision and individualize the therapeutic regimens by totally assuring patient’s cure under low cost standard basic dual therapy.

## Abbreviations

ADAR: adenosine deaminases acting on RNA
AFP: alfa-fetoprotein
ALT: alanine aminotransferase
AUROC: area under the operating curve
CBC: complete blood count
CHC: chronic hepatitis C
CYP2R1: cytochrome P-450 family 2, subfamily R
DAAs: direct acting antivirals
EASL: European Association for the Study of the Liver
ELISA: enzyme linked immunosorbent assay
G4: genotype 4
HALT-C: Hepatitis C Antiviral Long-term Treatment against Cirrhosis
HCV: hepatitis C virus
HOMA-IR: homeostasis model assessment of insulin resistance
IL28B: interleukin 28B
NR: non-responders
OASL: oligoadenylate synthetases-like
Peg-IFN: Pegylated interferon-alpha
ROC: receiving operating curve
RT-PCR: real time polymerase chain reaction
SNPs: single nucleotide polymorphisms
SVR: sustained viral response
VDR: vitamin D receptor
